# Comparative Evaluation of Fracture Resistance in Implant-Supported Provisional Crowns Fabricated by Computer-Aided Design and Manufacturing, Three-Dimensional Printing, and Conventional Self-Curing

**DOI:** 10.7759/cureus.86311

**Published:** 2025-06-18

**Authors:** Abhinav Shekhar, Akshim Rana, Shitij Srivastava, Love K Bhatia, Anshuman Chaturvedi, Abhishek Singh

**Affiliations:** 1 Department of Prosthodontics, Sardar Patel Post Graduate Institute of Dental and Medical Sciences, Lucknow, IND

**Keywords:** crowns, dental prosthesis, in vitro techniques, materials testing, prosthodontics

## Abstract

Aim and objectives

This study aimed to assess the influence of three fabrication techniques, computer-aided design (CAD) and computer-aided manufacturing (CAM) milling, three-dimensional (3D) printing, and conventional self-curing, on the fracture resistance of implant-supported polymethyl methacrylate (PMMA) resin-based provisional crowns.

Methods

An in vitro comparative study was conducted using 45 implant analogs with straight titanium abutments, equally distributed into three groups. Each analog was embedded vertically in custom acrylic resin blocks (20 × 20 × 20 mm). Abutments were torqued to 25 Ncm, and screw access channels were sealed with Teflon pellets and composite resin. Provisional crowns were fabricated using CAD-CAM milling, 3D printing, and conventional self-curing methods. All crowns were cemented and subjected to axial loading in a universal testing machine until fracture. Fracture resistance values were recorded and statistically analyzed.

Results

Crowns made with 3D printing showed the greatest average resistance to fracture, followed by CAD-CAM milling, and the crowns fabricated using the conventional self-curing method had the lowest fracture resistance. Statistical tests confirmed that these differences between the three groups were significant (p <0.05). The stronger performance of the 3D-printed crowns points to better overall durability, suggesting they could be especially useful for implant-supported provisional restorations in clinical practice.

Conclusion

Taking into account the limitations of this laboratory study, 3D-printed PMMA crowns showed the highest fracture resistance compared to the other fabrication methods tested. Crowns made by CAD-CAM milling also had strength levels that are acceptable for clinical use, making them a good alternative option. On the other hand, crowns produced with conventional self-curing methods were the least resistant to fracture, which means they might only be suitable for situations where the load is minimal. Ultimately, the choice of fabrication technique should be based on specific clinical requirements, material properties, and cost considerations.

## Introduction

In terms of implantology, provisional crown fabrication is a vital technique. Provisional crowns make an aesthetic contribution to the anterior aspect. They safeguard the operative site and provide patient comfort during the healing phase before the commencement of definitive prosthesis. Provisional crowns, however, should have specific mechanical properties, in particular strength and durability, for treatment to be successfully attained [1-2]. 

Self-curing polymethyl methacrylate (PMMA) resin has been popularly used as a material for the fabrication of provisional crowns and has been preferred because of its inexpensive nature and ease of manipulation [3]. However, with the advent of digital dentistry, new methods of fabrication, such as computer-aided design (CAD)/computer-aided manufacturing (CAM) milling and 3D printing, have been increasingly implemented. Thus, CAD-CAM milling produces crowns through subtractive machining of pre-polymerized PMMA blocks with high precision, while 3D printing fabricates crowns by an additive method through layer-wise polymerization of photoreactive resins. Both 3D printing and CAD-CAM milling have various benefits and drawbacks [4]. 

Despite the growing application of the technologies in prosthodontics, limited comparative data exist on the fracture resistance concerning conventional self-curing, CAD-CAM milling, and 3D printing of provisional crowns [5]. The absence of sufficient evidence may hinder clinicians from selecting an appropriate fabrication method, subsequently influencing the success rate from a clinical standpoint.

Accordingly, this study aims to evaluate and compare the fracture resistance of implant-supported provisional crowns fabricated using these three methods. The findings intend to provide clinicians with evidence-based guidance for optimizing provisional restoration selection and improving patient outcomes.

## Materials and methods

Ethical approval

The study complied with all ethical considerations outlined in the Declaration of Helsinki regarding the use of human-related materials. As no human or animal subjects were directly involved in the study, ethical clearance was granted by the Institutional Ethical Committee of Sardar Patel Post Graduate Institute of Dental and Medical Sciences, Lucknow, India (PROSTHO/02/222332/IEC). All procedures involving dental materials were conducted in accordance with the laboratory's safety and research guidelines.

Sample size and selection

A similar investigation reported mean fracture forces of 300.61 ± 98.94 N for conventional, 294.64 ± 60.34 N for CAD/CAM milled, and 408.49 ± 132.16 N for 3D-printed provisional prostheses [6].

Based on these results, the sample size was calculated using the formula [7], with the following parameters:

\[ n = \frac{1 + 1}{1} \times \left( \frac{291.44 \times 2.8}{334.58} \right)^2 = 11.89 \approx 12 \]

Considering a ratio of groups (r) as 1, a critical value of Zα/2 = 1.96 for 95% confidence, and Zβ = 0.84 for 80% power, with pooled SD = 291.44 and pooled mean difference d = 334.58. Applying these values to the formula, the calculated sample size was approximately 11.89, which was rounded to 12. To account for possible data loss (10%) and to maintain a balanced design with equal group sizes in multiples of three, the final proposed sample size was set at 15 samples per group.

Inclusion criteria for sample selection included commercially available implant analogs and titanium abutments compatible with standard implant systems. No exclusion criteria were applied as the study used standardized components. The sample size calculation was performed using standard statistical software.

Sample preparation

Implant analogs and straight titanium abutments (Noris Medical, Nesher, Israel) were vertically embedded in custom molds using self-cure acrylic resin (RR Cold Cure, Dental Products Of India Limited (DPI), Bangalore, India) to create uniform blocks measuring 20 × 20 × 20 mm. The abutments were torqued to the implant analogs at 20 Ncm. Screw access channels were sealed with polytetrafluoroethylene (PTFE) tape and composite resin (Filtek Z350 XT, 3M ESPE, Saint Paul, MN, USA). The operator was calibrated prior to sample fabrication to ensure consistency. Implant analogs and abutments were stored in a controlled environment at room temperature prior to embedding to prevent material degradation.

Standardization of crown dimensions

To ensure comparability between all groups, a standardized digital design of a first mandibular molar crown was created on the basis of ideal anatomical morphology. This digital model would be the work model for Groups A and B. Group A (CAD/CAM) printed the design directly from PMMA blanks via 5-axis milling. Meanwhile, Group B (3D printing) utilized the same digital design files for digital light processing (DLP) printing. For Group C (conventional self-curing), a wax pattern similar to the digital design in all dimensions was hand-sculpted with reference to the digital design so that external morphology and occlusal thickness (1.5 mm) would be uniform for all groups. Post fabrication, the dimensions and occlusal thickness of all crowns were verified using a digital caliper with 0.01 mm accuracy to ensure uniformity across groups.

Fabrication methods

Group A (CAD/CAM Milling)

Fifteen provisional crowns were fabricated with CAD/CAM milling from PMMA blanks (Telio CAD, Ivoclar Vivadent, Schaan, Liechtenstein). The crowns were designed and standardized according to the dimensions of the first mandibular molar, having an occlusal thickness of 1.5 mm. The design files remained the same for all samples, which were milled with a five-axis milling machine (DWX52D, DGSHAPE, Roland DGA, Irvine, CA, USA). Fabrication occurred at controlled room temperature (22 ± 2°C) by a single operator well versed with the technique to avoid discrepancies.

Group B (3D Printing)

Fifteen provisional crowns were fabricated by DLP 3D printing technology using PMMA-based photopolymer resin (Freeprint Temp, Detax GmbH, Ettlingen, Germany). The crowns had dimensions mimicking the first mandibular molar, as did Groups A and C. A DLP 3D printer with a 385-nm wavelength light source (NextDent 5100, 3D Systems, Rock Hill, SC, USA) was used to promote very good resin compatibility. Printing parameters were 100 microns layer thickness. After printing, the standard post-processing of all samples was performed: washing for 10 minutes in isopropyl alcohol and post-curing for 15 minutes in a UV-curing unit, according to the manufacturer's directions. Printing and post-processing were performed in controlled environmental conditions (22 ± 2°C, 50% humidity) by the same operator.

Group C (Conventional Self-Curing)

Fifteen provisional crowns were fabricated using autopolymerizing PMMA resin (DPI Cold Cure Acrylic Resin, DPI). A wax pattern replicating the dimensions of the first mandibular molar was prepared and used to create the mold space via the conventional flasking technique. After dewaxing, the acrylic resin powder and liquid were mixed according to the manufacturer's instructions and packed into the mold. The flask was closed and processed using the compression molding technique at room temperature (22 ± 2°C). Fabrication was performed by a single operator to maintain consistency.

**Figure 1:**
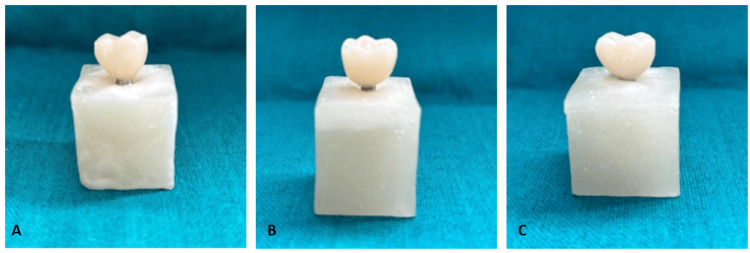
Representative image of provisional crowns (A) CAD/CAM milling; (B) 3D printing (DLP); and (C) conventional self-curing acrylic resin, showing standardized morphology and occlusal thickness CAD: computer-aided design; CAM: computer-aided manufacturing; DLP: digital light processing

Any crowns exhibiting visible defects or deviations from the specified dimensions were discarded and re-fabricated. No such rejections occurred in the present study. All fabrication procedures were carried out by a single trained operator to minimize variability (Figure 1).

Cementation and storage

All provisional crowns were tried on their respective implant abutments to ensure proper fit and adaptability. The crowns were luted to the abutments using non-eugenol temporary cement (RelyX Temp NE, 3M ESPE) following the manufacturer's guidelines. Excess cement was carefully removed with an explorer and cotton pellets. After cementation, the samples were stored in distilled water at 37°C for 24 hours prior to mechanical testing to simulate oral conditions.

A standardized seating force of 50 N was applied during cementation using a mechanical press to ensure uniform luting pressure. Storage water was changed every 12 hours, and temperature was continuously monitored to maintain 37 ± 1°C.

Aging of samples

In order to simulate the thermal stresses encountered in the oral environment, all specimens were subjected to a standardized thermocycling protocol in compliance with International Organization for Standardization (ISO)/TS 11405. This procedure entailed alternating immersion in distilled water baths maintained at 5°C and 55°C, with each temperature exposure lasting 13 seconds, for a total of 30,000 cycles. Upon completion of thermocycling, the specimens were equilibrated to room temperature prior to mechanical testing.

Testing method

The fracture resistance for all provisional crowns was measured by a universal testing machine (Instron Model 3382, Instron Industrial Products, Norwood, MA, USA). An axial load was applied at a crosshead speed of 1 mm/min, with the use of a rubber dam to ensure homogeneous distribution of force across the system. The maximal force at fracture was obtained for analysis.

**Figure 2:**
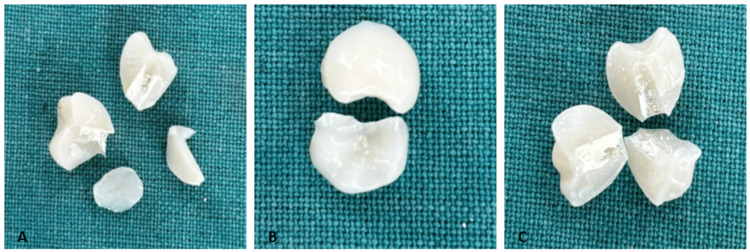
Representative image of fractured provisional crowns (A) CAD/CAM milling; (B) 3D printing (DLP); and (C) conventional self-curing acrylic resin groups, showing typical fracture fragments after mechanical testing CAD: computer-aided design; CAM: computer-aided manufacturing; DLP: digital light processing

The fracture load was applied using a spherical stainless-steel indenter of 5 mm diameter positioned at the central occlusal fossa to mimic clinical loading conditions. No preload or cyclic fatigue testing was performed. The operator conducting the tests was blinded to the group allocation to reduce bias (Figure 2).

Data collection and statistical analysis

A fracture resistance test was carried out using a universal testing machine, applying an axial load and recording the maximum force at fracture. Data on fracture resistance values were entered into a Microsoft Excel sheet (Microsoft Corp., Redmond, WA, USA) for analysis using IBM SPSS Statistics software, version 21 (IBM Corp., Armonk, NY, USA) with descriptive statistics and analysis of variance (ANOVA) used to compare the results produced by different fabrication methods.

The quantitative data, such as measurements of fracture strength, were summarized using descriptive statistics, including mean, median, and standard deviations (SDs). For comparisons of fracture resistance among groups of different fabrication procedures, a one-way analysis of variance was implemented to assess for differences among groups. If differences proved to be significant, post-hoc testing was performed using Tukey’s honest significant difference (HSD) test to establish between which groups the differences lay. Intergroup changes with time or between different conditions were also scrutinized by Tukey’s HSD test. At pairwise comparisons, the results showed very significant interpretations, with p-values below 0.001, thereby strongly rejecting the null hypothesis. A p-value of less than 0.05 was chosen to represent significance for all tests.

## Results

Distribution of 45 implant-supported provisional crowns was divided into three equal groups based on their fabrication methods (Table 1).

**Table 1:** Distribution of implant-supported provisional crowns according to the groups Percentages may not total exactly 100% due to rounding; CAD: computer-aided design; CAM: computer-aided manufacturing

S. No.	Group	Fabrication method	Sample (n)	Percentage (%)
1	Group A	CAD-CAM	15	33.3
2	Group B	3D printing	15	33.3
3	Group C	Conventional self-curing	15	33.3
	Total		45	100.0

Among the CAD-CAM-fabricated implant-supported provisional crowns, the minimum fracture strength was 430.0 N, and the maximum fracture resistance was 465.0 N. The mean fracture resistance of the implant-supported provisional crowns fabricated by the CAD-CAM process was 455.93±8.13 N (Table 2).

**Table 2:** Fracture resistance of implant-supported provisional crowns fabricated by different methods The table summarizes the minimum, maximum, mean, median, and standard deviation (SD) values for fracture resistance measured across three fabrication techniques: computer-aided design (CAD)-computer-aided manufacturing (CAM) milling (Group A), 3D printing (Group B), and conventional self-curing (Group C).

Group	Minimum	Maximum	Mean	Median	SD
Group A	430	465	455.93	457	8.13
						Group B	600	658	607.2	604	14.23
Group C	400	432	414.8	415	7.12						

Among the implant-supported provisional crowns fabricated by the 3D printing technique, the minimum fracture resistance was 600.0 N, and the maximum fracture resistance was 658.0 N. The mean fracture resistance of the 3D-printed implant-supported provisional crowns was 607.20±14.23 N (Table 2).

Among the implant-supported provisional crowns fabricated by the conventional self-curing method, the minimum fracture resistance was 400.0 N, and the maximum fracture resistance was 432.0 N. The mean fracture resistance of the conventionally fabricated implant-supported provisional crowns was 414.80±7.12 N (Table 2).

**Figure 3:**
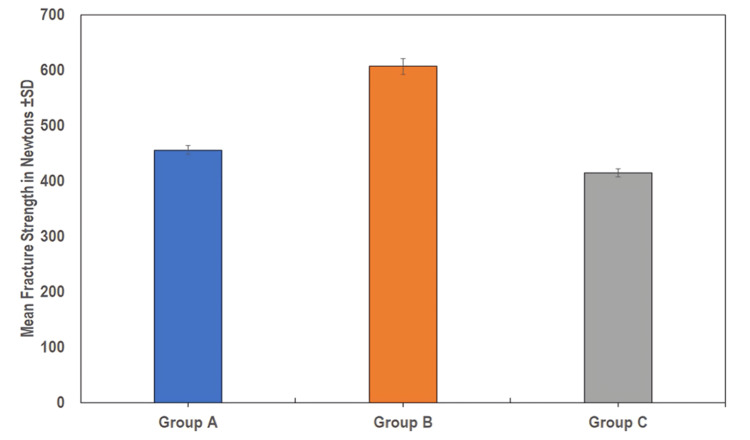
Intergroup comparison of fracture resistance (N) among provisional crowns (Group A, Group B, and Group C) Group A: computer-aided design (CAD)-computer-aided manufacturing (CAM) milling; Group B: 3D printing; Group C: conventional self-curing

The mean fracture resistance of the implant-supported provisional crowns fabricated by the CAD-CAM process was 455.93±8.13 N. The mean fracture resistance of the 3D-printed implant-supported provisional crowns was 607.20±14.26 N. The mean fracture resistance of the conventionally fabricated implant-supported provisional crowns was 414.80±7.12 N. On comparing statistically, the fracture resistance of the 3D-printed implant-supported provisional crowns (Group B) was significantly higher as compared to those fabricated by CAD-CAM (Group A) and conventionally fabricated (Group C) implant-supported provisional crowns (F=1442.988; p<0.001) (Table 3; Figure 3).

**Table 3:** Intergroup comparison of fracture resistance The table presents the mean and standard deviation (SD) of fracture resistance for Group A (computer-aided design (CAD)-computer-aided manufacturing (CAM) milling), Group B (3D printing), and Group C (conventional self-curing). The ANOVA test results, including the F-value and significance level (p-value), indicate statistically significant differences among the groups. Statistically significant differences are indicated by p < 0.001.

S. No.	Group	Fracture resistance (N)		ANOVA	
		Mean	SD	F	‘p’
1	Group A	455.93	8.13	1442.988	<0.001
2	Group B	607.20	14.26		
3	Group C	414.80	7.12		

On comparing statistically (paired group), a significantly higher fracture resistance was found for 3D printing implant-supported provisional crowns (Group B) as compared to CAD-CAM milling (Group A) and conventional self-curing (Group C). Further, a significantly higher fracture resistance was also found in group A as compared to group C (p<0.001) (Table 4).

**Table 4:** Between-group comparison of fracture resistance Comparison of mean differences in fracture resistance between groups A, B, and C. Values represent the mean difference, standard error (SE), p-value, 95% confidence interval (CI), and q-statistic from the Tukey's honest significant difference (HSD) post-hoc test following one-way ANOVA. The identical SE values across comparisons reflect the pooled standard error used by the Tukey HSD test based on the within-group variability. Statistically significant differences are indicated by p < 0.001.

Comparison	Mean Difference (N)	SE	p-value	95% CI (Lower–Upper)	q-statistic
Group A vs. Group B	-151.26	3.77	<0.001	-160.43 to -142.10	39.51
Group A vs. Group C	41.13	3.77	<0.001	+31.97 to +50.30	10.74
Group B vs. Group C	192.4	3.77	<0.001	+183.24 to +201.56	50.25

## Discussion

The objective of this in vitro investigation was to compare the fracture resistance of implant-supported provisional crowns fabricated by three techniques: CAD-CAM milling, 3D printing, and conventional self-curing techniques. Statistically significant differences were observed between groups, with the highest fracture resistance exhibited by the 3D-printed crowns and a progressively lesser resistance rate for the CAD-CAM and conventional crowns, respectively.

Among the conclusions based on the study is that 3D-printed provisional crowns indeed can perform better, as this improved strength can be attributed to additive layering, which is controlled more strictly, and resin formulation, along with the enhanced internal design configuration considered during manufacturing processes. Therefore, these factors could render 3D printing highly appropriate for high-load provisional restoration [8-9]. However, it must be noted that 3D-printed resins exhibit anisotropic properties, where mechanical strength may vary depending on build orientation. This variable was not controlled in the present study and warrants further exploration, as different orientations may impact clinical reliability.

The CAD-CAM milled crowns yielded moderate fracture resistance, probably a result of a homogeneous resin block and an accurate digital fabrication. Its strength and consistency, though lesser than that of the 3D-printed ones, are still sufficient for clinical use, standing up to the assertions of previous literature, which found them able to be reliably employed in provisional use [10-11]. As per existing evidence, CAD-CAM crowns often demonstrate high fracture resistance in laboratory settings, though these findings may not directly translate to the oral environment without simulated aging and functional loading.

Fracture resistance for conventionally fabricated crowns was lowest, probably because of the porosity of materials, errors due to manual processing, and lesser control of polymerization. Conventional techniques with their mechanical limitations continue to be practiced primarily [12].

While this study supports the notion of mechanical superiority of 3D-printed provisional crowns, other studies suggest that there would be tooth type, margin design, or printer/material-dependent differences in fracture strength [13]. Such differences draw attention to the concern that both mechanical and clinical features play important roles in the choice of fabrication method. Furthermore, it should be emphasized that static fracture testing, as performed in this study, provides only a limited snapshot of material performance. Intraoral conditions are far more complex, involving thermal fluctuations, masticatory fatigue, and multidirectional loading. Future research should incorporate simulated aging protocols such as cyclic loading and thermomechanical stress to better approximate real-world outcomes. Moreover, the influence of the luting agent, its type, thickness, and application, is standardized in this study but remains a critical variable that may significantly affect clinical performance.

Clinical implications of the study

Clinical selection of any transfer technique for the fabrication of an implant-supported provisional crown is important because of the results of this in vitro investigation. The significantly higher fracture resistance of 3D-printed crowns indicates their application in areas of high stress or in posterior regions where mechanical strength is paramount. On the other hand, 3D printing and CAD-CAM milling provide benefits such as digital accuracy, repeatability, and efficient workflow that can thus optimize chairside time and other treatment outcomes. The conventional self-curing methods are arguably less reliable when prolonged function and higher resistance to load are required; therefore, those methods have remained a crumb of the commonly used methods owing to cost and accessibility. The results indicate a more frequent recommendation by clinicians of digital fabrication techniques in the construction of provisional restorations, especially in implant-supported ones. However, since making the final treatment decision involves considering patient-specific factors such as esthetic demands, economic factors, and clinical setting, more in vivo studies should follow to validate these findings and to assess long-term clinical performance in dynamic oral conditions.

Limitations of the study

This in vitro study does not fully replicate the complex oral environment, as factors like dynamic occlusal loading, humidity, salivary enzymes, and long-term fatigue were not simulated. While thermocycling was performed, additional aging protocols such as mechanical loading and water storage were not included.

Simplified crown forms were used for standardization, which may not reflect the true anatomical complexity and stress distribution in clinical scenarios. Each group tested only one material and design workflow, limiting generalizability across the broader spectrum of available systems.

The influence of the luting agent was kept constant, and the anisotropic effects of print orientation in 3D-printed crowns were not evaluated. Though the sample size met statistical requirements, it may not fully reflect clinical variability. Future studies should incorporate in vivo conditions, varied materials, and patient-centered outcomes to enhance clinical relevance.

## Conclusions

Within the boundaries of this in vitro study, it can be concluded that there is a significant effect of fabrication method on the fracture resistance of implant-supported provisional crowns. 3D printing showed the highest fracture resistance, followed by CAD-CAM milling, and finally, conventional self-cure among the three fabrication methods. These findings emphasize how promising additive manufacturing could be for making strong provisional restorations, especially in load-bearing areas. Decision-making in clinical practice, however, will consider other factors such as cost, availability, aesthetics, and specifics of the case. More in vivo studies need to be done to ascertain these results under functional intraoral conditions and to evaluate the long-term durability of a provisional crown fabricated digitally.
